# Protection Mechanism of *Clostridium butyricum* against *Salmonella* Enteritidis Infection in Broilers

**DOI:** 10.3389/fmicb.2017.01523

**Published:** 2017-08-09

**Authors:** Xiaonan Zhao, Jie Yang, Lili Wang, Hai Lin, Shuhong Sun

**Affiliations:** College of Animal Science and Technology, Shandong Agricultural University Tai’an, China

**Keywords:** AA broilers, oral administration, *S.* enteritidis, *C. butyricum*, Q-PCR

## Abstract

This study was designed to evaluate the protection mechanism of oral administration of *Clostridium butyricum* against *Salmonella* enteritidis (SE) colonization in broilers. In the current study, 180 one-day-old healthy Arbor Acres (AA) broilers were meanly grouped into three, with three replicates of 20 birds each. An negative control group was fed basal diet without SE challenge and a positive control (PC) group was fed the basal diet and challenged with SE [10^6^ colony forming unit (CFU)/0.2 mL]. An experimental (EXP) group was fed the basal diet, orally administered with *C. butyricum* (10^6^ CFU/mL) and challenged with SE (10^6^ CFU/0.2 mL). The results showed that compared to the PC group, the SE loads in livers, spleens, and cecal contents of chickens in EXP group were significantly reduced (*P* < 0.05) except in spleens at the 2-day post-infection; the production of interferon-γ, interleukin (IL)-1β, IL-8, and tumor necrosis factor-α in the livers, spleens, and cecal tissues of chickens in EXP group were decreased to different extents. The results of quantitative real-time polymerase chain reaction further revealed that the inflammation of chickens in EXP group was alleviated by *C. butyricum* via down-regulating TLR4, MyD88, and NF-κB-dependent pathways. Collectively, these findings indicated that oral administration of *C. butyricum* could be a suitable alternative for preventing SE infection in broilers.

## Introduction

*Salmonella*, as an important foodborne pathogen, can lead to serious infections in animals and humans worldwide (Mead et al., 1999; Scallan et al., 2011). Poultry have been recognized as an important reservoir for *Salmonella* (Chen and Jiang, 2014). *Salmonella* can cause high morbidity and mortality in poultry breeding industry, especially in young birds within 1 week age (Wigley et al., 2001; Vo et al., 2006). At the early stage of *Salmonella* infection, the production of cytokines, such as interferon (IFN)-γ, interleukin (IL)-1β, IL-8, and tumor necrosis factor (TNF)-α, is of utmost importance for controlling *Salmonella* growth and spread in the host body (Brown et al., 2006; Hu et al., 2015). In addition, Toll-like receptors (TLRs) can play a key role in the protection animals and humans against *Salmonella* infection, and they combat the pathogen through recognition of pathogen-associated molecular patterns (Akira and Takeda, 2004). TLR4, as one important member of the TLRs family, can recognize lipopolysaccharide (LPS) of Gram-negative bacteria and can activate nuclear factor-kappa B (NF-κB) through myeloid differentiation primary response protein 88 (MyD88), and therefore leading to cytokine secretion and inflammatory response (Kawai and Akira, 2007).

As for the combat against *Salmonella* infections, antimicrobials have been widely used in the clinical practice. However, the overuse and even abuse of antibiotics have contributed to the increasing and dissemination of drug-resistant *Salmonella* and have sparked a severe public health concern (Chiu et al., 2002; Tseng et al., 2014). Furthermore, antimicrobials can lead to the loss of commensal gastrointestinal microbiota and potentially to the overgrowth of pathogens (McDonald et al., 2016; Wischmeyer et al., 2016). Therefore, in the recent years, many researchers have been striving to find the substitutes of antimicrobials, and probiotics are being considered as one of the promising substitute for antimicrobials against *Salmonella* infections (Mathipa and Thantsha, 2017).

Probiotics have ability to provide protection effects for the host when administered in adequate amounts (Food and Agricultural Organization/World Health Organization [FAO/WHO], 2002). Numerous studies have showed that the use of probiotics is able to modulate mucosal immune functions, prevent bacterial translocation, and potentially suppress inflammatory cytokine production through modulating LPS-induced inflammation by binding to LPS or directly perturbing the MyD88 signaling pathway (Mainous et al., 1995; Kemgang et al., 2014).

*Clostridium butyricum*, a strictly anaerobic endospore-forming Gram-positive bacillus, could produce butyric acid. Compared to *Lactobacillus* and *Bifidobacterium*, *C. butyricum* is able to survive at lower pH and relatively higher bile concentrations (Okamoto et al., 2000; Zhang et al., 2016). Previous studies demonstrated that *C. butyricum* can inhibit pathogens propagation and spread in host body and therefore is considered as a potential substitute for antibiotics (Gao et al., 2012; Yang et al., 2012; Zhang et al., 2016). However, the protection mechanism of *C. butyricum* against *Salmonella* enteritidis (SE) colonization in broilers remains to be elucidated. This study was therefore conducted to better understand the protection mechanism by which *C. butyricum* protects chickens against SE infection.

## Materials and Methods

### Ethics Statement

All procedures were approved by the Animal Care and Use committee of Shandong Agricultural University (SDAUA-2016-016).

### Bacterial Strains and Growth Conditions

*Clostridium butyricum* (AQQF01000149) was obtained as a gift from Dalian Sanyi Animal Medicine Company (China) and grown anaerobically at 37°C in liquid fermentation tank for 48 h. The concentration of *C. butyricum* was adjusted to 1 × 10^6^ colony forming unit (CFU)/mL in sterile saline.

A virulent atrichia SE, a isolate from a diseased chicken, was obtained from the Avian Disease Centre of Shandong Agricultural University. While cultivating SE, single colony was picked from xylose lysine deoxycholate agar plate and transferred into a tube contained 5 mL tryptic soy broth and then incubated at 37°C for 12 h. The concentration of SE was adjusted to 1 × 10^6^ CFU/mL in sterile saline.

### Experimental Design

The experiment was performed in October, 2016. In total, 180 one-day-old healthy Arbor Acres (AA) chickens (negative for *Salmonella*) were bought from a hatchery in Xintai, China. Chickens were housed in metal cages and provided *ad libitum* with water and commercial starter diet in the animal room of Shandong Agricultural University. The temperature was maintained at 30°C at the first 3 days and gradually reduced to 28°C during the last days of the experiment. The nutrient levels of the basal diets met the nutritional requirement of the broilers (NRC, 1994) (**Table 1**), and the rearing duration lasted 2 weeks. The sanitation of raising environments were regularly cleaned for the health of chicken. Chickens were divided into three treatment groups in random manner: an negative control (NC) group, chickens were orally administrated 0.2 mL sterile saline per chick once every day through day 1 to day 7; a positive control (PC) group, chickens were challenged with 0.2 mL SE enrichment solution (10^6^ CFU/0.2 mL) at the 8 day, and were given sterile saline (0.2 mL/chick) during day 1 to day 7; an experimental (EXP) group, chickens were given 0.2 mL *C. butyricum* enrichment solution (10^6^ CFU/0.2 mL) once every day from 1 to 7 day, and at the 8 day, chickens were challenged with 0.2 mL SE enrichment solution (10^6^ CFU/0.2 mL). For all groups, chickens were euthanized via cervical dislocation, and livers, spleens, as well as cecal tissues and contents were sampled at 2 and 6 days of post-infection. These samples were frozen at -80°C for further analyses.

**Table 1:** The composition and nutrients of basal diet.

Ingredient	Content (%)	Chemical composition	Content
Corn	55.23	CP, %	20.90
Soybean meal	30.67	ME, Mcal/kg	3.00
Wheat shorts	4.00	Calcium, %	1.00
Fish meal^a^	3.00	Total P, %	0.65
Soybean oil^b^	2.90	Available P, %	0.45
DL-methionine	0.27	Methionine + cysteine, %	0.90
NaCl	0.27	Lysine, %	1.05
Limestone	1.33		
Calcium phosphate	1.33		
Vitamin–mineral premix^c^	1.00		


*^a^Crude protein content is 62.5% and metabolizable energy is 2.79 Mcal/kg.*

*^b^Metabolizable energy is 8.8 Mcal/kg. ^c^Supplied per kilogram of diet: vitamin A (retinyl acetate), 1,500 IU; cholecalciferol, 200 IU; vitamin E (DL-α-tocopheryl acetate), 10 IU; riboflavin, 3.5 mg; pantothenic acid, 10 mg; niacin, 30 mg; cobalamin, 10 μg; choline chloride, 1,000 mg; biotin, 0.15 mg; folic acid, 0.5 mg; thiamine 1.5 mg; pyridoxine 3.0 mg; Fe, 80 mg; Zn, 40 mg; Mn, 60 mg; I, 0.18 mg; Cu, 8 mg; Se, 0.15 mg.*

### SE Translocation

SE translocation to livers, spleens, and cecal contents of all SE-challenged groups was determined at 2 and 6 days of post-infection. Livers, spleens, and cecal contents were weighted, homogenized respectively and serially diluted 10-fold with sterile phosphate-buffered saline (1:10, w/v), and then screened on Brilliant Green Agar plates (Hopebio, Qingdao, China) to count the CFU of SE after incubation at 37°C for 24 h.

### Quantitative Real-time Polymerase Chain Reaction

Quantitative real-time polymerase chain reaction (Q-PCR) was undertaken to relatively quantify the expression levels of cytokine genes including IFN-γ, IL-1β, IL-8, and TNF-α, and the gene expressions of the MyD88-dependent pathway of TLR4, MyD88, and NF-κB in the livers, spleens, and cecal tissues. At 2 and 6 days of post-infection, Trizol reagent (Invitrogen) was used to extract total RNA from livers, spleens, and cecal tissues according to the manufacturer’s instruction. Nano Drop 2000 spectrophotometer (Thermo Fisher Scientific, MA, United States) was used to determine the concentration and quality of total RNA. SuperScript III First Strand synthesis kit (Life Technologies, Carlsbad, CA, United States) was used to synthesize cDNA with 2 μg of total RNA. The cDNA was stored at -20°C. The Q-PCR was performed with SYBR Green master mix using 7500 Fast Real-Time PCR system (Applied Biosystems, Carlsbad, CA, United States). PCR conditions contained one cycle of 95°C for 30 s, followed by 40 cycles of 95°C for 5 s and 60°C for 34 s. Dissociation analysis of amplification products was performed at the end of each PCR to confirm the specificity of amplicon. The primers for real-time PCR are listed in **Table 2**. mRNA relative expression was calculated using the 2^-ΔΔCt^ method.

**Table 2:** Primers for Q-PCR in this study.

Gene	Sequence (5′–3′)	GenBank No.
TLR4	Forward: AGTCTGAAATTGCTGAGCTCAAAT	AY064697
	Reverse: GCGACGTTAAGCCATGGAAG	
MyD88	Forward: TGATGCCTTCATCTGCTACTG	EF011109
	Reverse: TCCCTCCGACACCTTCTTTCTA	
NF-κB	Forward: CAGCCCATCTATGACAACCG	NM_-_205129
	Reverse: TCCCTGCGTCTCCTCTGTGA	
IFN-γ	Forward: ATCATACTGAGCCAGATTGTTTC	NM_-_205149.1
	Reverse: ATCATACTGAGCCAGATTGTTTC	
IL-1β	Forward: GTGAGGCTCAACATTGCGCTGTA	Y15006
	Reverse: TGTCCAGGCGGTAGAAGATGAAG	
IL-8	Forward: ATGAACGGCAAGCTTGGAGCTG	AJ009800
	Reverse: TCCAAGCACACCTCTCTTCCATCC	
TNF-α	Forward: TGCTGTTCTATGACCGCC	AY765397
	Reverse: CTTTCAGAGCATCAACGCA	
β-Actin	Forward: GAGAAATTGTGCGTGACATCAy	L08165
	Reverse: CCTGAACCTCTCATTGCCA	


### Statistical Analysis

The one-way ANOVA and Student’s *t*-test of SPSS 15.0 (SPSS Inc., Chicago, IL, United States) were used to perform statistical analyses. The results were shown as mean ± standard deviations (SD). Differences were considered significant at *P* < 0.05.

## Results

### SE Translocation

The results of SE translocation showed that after 2 and 6 days post-infection, chickens in EXP group significantly reduced the viable count of SE compared to the PC group in the liver, spleen, and cecal content (*P* < 0.05), except in the spleen at 2 day post-infection (*P* > 0.05). In addition, SE was not detected in NC group (**Table 3**).

**Table 3:** Effect of *C. butyricum* on the reduction of SE counts in livers, spleens, and cecal contents of broilers^1^.

	Liver		Spleen		Cecal content	
			
	Log CFU/organ		Log CFU/organ		Log CFU/organ	
			
Item	2 d post-ch^2^	6 d post-ch	2 d post-ch	6 d post-ch	2 d post-ch	6 d post-ch
PC	1.78 ± 0.29^a^	1.72 ± 0.44^a^	0.72 ± 0.47	0.63 ± 0.32^a^	5.82 ± 0.86^a^	5.48 ± 0.71^a^
EXP	ND^b^	ND^b^	0.42 ± 0.20	ND^b^	0.86 ± 0.12^b^	0.66 ± 0.12^b^


*Mean ± SD in the same line with different superscript letters differ significantly (*P* < 0.05). ^1^Each mean represents six birds. PC, birds fed a basal diet and challenged with SE; EXP, birds fed a basal diet with *C. butyricum* (10^*6*^ CFU/mL) and challenged with SE. Results show the colony counts of SE in different organs, they are expressed as mean (Log_*10*_ CFU/g of organ) ± SD. ^2^The days after challenging.*

### Gene Expression of Cytokines in the Liver

At 2-day post-infection, gene expression for pro-inflammatory cytokine TNF-α was significantly elevated in PC group compared to NC and EXP groups (*P* < 0.05), but no significant difference was found between NC and EXP groups (*P* > 0.05); with regard to IFN-γ, IL-1β, and IL-8 production, no significant difference was observed among EXP, PC, and NC groups (*P* > 0.05). At 6-day post-infection in the PC group, the gene expressions of IFN-γ, IL-1β, and TNF-α were elevated significantly (*P* < 0.05) compared to NC and EXP groups, but no difference was found between NC and EXP groups (*P* > 0.05); in terms of IL-8, no significant differences were found among EXP, NC, and PC groups (*P* > 0.05) (**Table 4**).

**Table 4:** Fold changes of cytokine gene expression in the livers of broilers after challenged with SE^1^.

		Experimental treats		
		
Gene	Age of post-ch^2^	NC	PC	EXP
IFN-γ	2 d	0.001 ± 0.0001	0.001 ± 0.0001	0.0007 ± 0.0001
	6 d	0.60 ± 0.15^b^	1.36 ± 0.16^a^	0.49 ± 0.04^b^
IL-1β	2 d	0.64 ± 0.19	0.71 ± 0.13	0.89 ± 0.18
	6 d	0.55 ± 0.05^b^	1.09 ± 0.12^a^	0.31 ± 0.03^b^
IL-8	2 d	0.34 ± 0.06	0.78 ± 0.11	0.80 ± 0.21
	6 d	0.59 ± 0.19	1.39 ± 0.21	0.76 ± 0.28
TNF-α	2 d	0.56 ± 0.06^b^	0.92 ± 0.18^a^	0.30 ± 0.14^b^
	6 d	0.83 ± 0.06^b^	2.51 ± 0.32^a^	1.00 ± 0.08^b^


*Mean ± SD in the same row with different superscript letters differ significantly (*P* < 0.05). ^1^Each mean represents six birds. NC, birds fed a basal diet without challenged with SE; PC, birds fed a basal diet and challenged with SE; EXP, birds fed a basal diet with *C. butyricum* (10^*6*^ CFU/mL) and challenged with SE. ^2^The days after challenging.*

### Gene Expression of Cytokines in the Spleen

At 2-day post-infection, gene expression for IFN-γ was significantly increased in PC group compared to NC and EXP groups (*P* < 0.05), but no significant difference was found between NC and EXP groups (*P* > 0.05); in addition, no significant differences were observed in the IL-1β and TNF-α productions among EXP, PC, and NC groups (*P* > 0.05); the expression of IL-8 was significantly mounted in the PC group compared to EXP group (*P* < 0.05), but no significant difference was observed between EXP and NC groups (*P* > 0.05), and the same change was found between PC and NC groups (*P* > 0.05). At 6-day post-infection, gene expressions of IFN-γ, IL-1β, and IL-8 were elevated significantly in the PC group compared to NC and EXP groups (*P* < 0.05), but no significant difference was found between NC and EXP groups (*P* > 0.05); additionally, no significant difference in the TNF-α production was found among EXP, NC, and PC groups (*P* > 0.05) (**Table 5**).

**Table 5:** Fold changes of cytokine gene expression in the spleens of broilers after challenged with SE^1^.

		Experimental treats		
		
Gene	Age of post-ch^2^	NC	PC	EXP
IFN-γ	2 d	1.00 ± 0.35^b^	2.30 ± 0.19^a^	1.29 ± 0.12^b^
	6 d	0.39 ± 0.03^b^	1.31 ± 0.22^a^	0.38 ± 0.01^b^
IL-1β	2 d	0.99 ± 0.15	2.19 ± 0.36	1.17 ± 0.34
	6 d	0.63 ± 0.14^b^	1.64 ± 0.22^a^	0.32 ± 0.08^b^
IL-8	2 d	0.58 ± 0.22^ab^	1.81 ± 0.28^a^	0.30 ± 0.18^b^
	6 d	0.57 ± 0.09^b^	1.95 ± 0.24^a^	0.79 ± 0.12^b^
TNF-α	2 d	0.83 ± 0.11	0.80 ± 0.10	1.37 ± 0.49
	6 d	0.008 ± 0.0008	0.009 ± 0.001	0.007 ± 0.001


*Mean ± SD in the same row with different superscript letters differ significantly (*P* < 0.05). ^1^Each mean represents six birds. NC, birds fed a basal diet without challenged with SE; PC, birds fed a basal diet and challenged with SE; EXP, birds fed a basal diet with *C. butyricum* (10^*6*^ CFU/mL) and challenged with SE. ^2^The days after challenging.*

### Gene Expression of Cytokines in the Cecal Tissues

At 2-day post-infection, gene expression for IL-1β was significantly elevated in PC group compared to NC and EXP groups (*P* < 0.05), but no significant difference was found between NC and EXP groups (*P* > 0.05); with regard to IFN-γ, IL-8, and TNF-α production, no significant difference was observed among EXP, PC, and NC groups (*P* > 0.05). At 6-day post-infection, gene expressions of IFN-γ, IL-1β, and IL-8 were elevated significantly in PC group compared to NC and EXP groups (*P* < 0.05), but no significant difference was found between NC and EXP groups (*P* > 0.05); of note, no significant difference in the TNF-α was found among EXP, NC, and PC groups (*P* > 0.05) (**Table 6**).

**Table 6:** Fold changes of cytokine gene expression in the cecal tissues of broilers after challenged with SE^1^.

		Experimental treats		
		
Gene	Age of post-ch^2^	NC	PC	EXP
IFN-γ	2 d	0.62 ± 0.11	1.33 ± 0.17	0.70 ± 0.22
	6 d	0.49 ± 0.07^b^	1.37 ± 0.22^a^	0.56 ± 0.15^b^
IL-1β	2 d	0.80 ± 0.12^b^	1.21 ± 0.14^a^	0.48 ± 0.11^b^
	6 d	0.66 ± 0.17^b^	1.29 ± 0.16^a^	0.54 ± 0.12^b^
IL-8	2 d	0.67 ± 0.04	0.44 ± 0.10	0.34 ± 0.09
	6 d	0.66 ± 0.15^b^	1.41 ± 0.16^a^	0.50 ± 0.06^b^
TNF-α	2 d	1.00 ± 0.08	0.75 ± 0.17	0.89 ± 0.18
	6 d	0.93 ± 0.08	0.76 ± 0.17	0.89 ± 0.18


*Mean ± SD in the same row with different superscript letters differ significantly (*P* < 0.05). ^1^Each mean represents six birds. NC, birds fed a basal diet without challenged with SE; PC, birds fed a basal diet and challenged with SE; EXP, birds fed a basal diet with *C. butyricum* (10^*6*^ CFU/mL) and challenged with SE. ^2^The days after challenging.*

### Expression of Genes of the MyD88-Dependent Pathway in Liver, Spleen, and Cecal Tissues

At 2-day post-infection, no significant difference was observed between EXP and PC groups with regard to the production of TLR4, MyD88, and NF-κB in liver, spleen, and cecal tissues (*P* > 0.05). However, at 6-day post-infection, gene expressions for TLR4, MyD88, and NF-κB in liver, spleen, and cecal tissues were elevated significantly (*P* < 0.05) in the PC group compared to NC and EXP groups (*P* < 0.05), but no significant differences were found between EXP and NC groups (*P* > 0.05) (**Tables 7**–**9**).

**Table 7:** Expression of genes of the MyD88-dependent pathway in livers^1^.

		Experimental treats		
		
Gene	Age of post-ch^2^	NC	PC	EXP
TLR4	2 d	0.78 ± 0.27	1.21 ± 0.25	0.99 ± 0.24
	6 d	0.81 ± 0.23^b^	2.48 ± 0.38^a^	1.35 ± 0.28^b^
MyD88	2 d	0.66 ± 0.09	1.01 ± 0.07	0.63 ± 0.03
	6 d	0.81 ± 0.19^b^	1.65 ± 0.22^a^	0.70 ± 0.10^b^
NF-κB	2 d	0.41 ± 0.04	0.62 ± 0.10	0.57 ± 0.10
	6 d	0.57 ± 0.06^b^	1.18 ± 0.11^a^	0.41 ± 0.01^b^


*Mean ± SD in the same row with different superscript letters differ significantly (*P* < 0.05). ^1^Each mean represents six birds. NC, birds fed a basal diet without challenged with SE; PC, birds fed a basal diet and challenged with SE; EXP, birds fed a basal diet with *C. butyricum* (10^*6*^ CFU/mL) and challenged with SE. ^2^The days after challenging.*

**Table 8:** Expression of genes of the MyD88-dependent pathway in spleens^1^.

		Experimental treats		
		
Gene	Age of post-ch^2^	NC	PC	EXP
TLR4	2 d	0.75 ± 0.08	0.85 ± 0.05	0.93 ± 0.09
	6 d	0.78 ± 0.18^b^	1.59 ± 0.17^a^	0.66 ± 0.09^b^
MyD88	2 d	1.01 ± 0.07	1.05 ± 0.07	0.91 ± 0.04
	6 d	0.80 ± 0.05^b^	1.46 ± 0.15^a^	0.81 ± 0.07^b^
NF-κB	2 d	1.00 ± 0.67	0.91 ± 0.53	0.84 ± 0.55
	6 d	0.65 ± 0.07^b^	1.54 ± 0.15^a^	0.62 ± 0.13^b^


*Mean ± SD in the same row with different superscript letters differ significantly (*P* < 0.05). ^1^Each mean represents six birds. NC, birds fed a basal diet without challenged with SE; PC, birds fed a basal diet and challenged with SE; EXP, birds fed a basal diet with *C. butyricum* (10^*6*^ CFU/mL) and challenged with SE. ^2^The days after challenging.*

**Table 9:** Expression of genes of the MyD88-dependent pathway in cecal tissues^1^.

		Experimental treats		
		
Gene	Age of post-ch^2^	NC	PC	EXP
TLR4	2 d	0.72 ± 0.12	0.86 ± 0.15	0.75 ± 0.14
	6 d	0.93 ± 0.05^b^	2.15 ± 0.23^a^	0.81 ± 0.11^b^
MyD88	2 d	0.52 ± 0.08^b^	0.92 ± 0.06^a^	0.91 ± 0.07^a^
	6 d	0.95 ± 0.05^b^	1.59 ± 0.15^a^	0.46 ± 0.15^b^
NF-κB	2 d	0.92 ± 0.26	1.22 ± 0.82	0.97 ± 0.34
	6 d	0.83 ± 0.07^b^	1.61 ± 0.13^a^	0.74 ± 0.15^b^


*Mean ± SD in the same row with different superscript letters differ significantly (*P* < 0.05). ^1^Each mean represents six birds. NC, birds fed a basal diet without challenged with SE; PC, birds fed a basal diet and challenged with SE; EXP, birds fed a basal diet with *C. butyricum* (10^*6*^ CFU/mL) and challenged with SE. ^2^The days after challenging.*

## Discussion

In the present study, compared with the PC group, the levels of SE recovered from liver, spleen, and cecal contents were reduced in 1-day-old chickens fed *C. butyricum* for seven consecutive days, which was in agreement with previous reports (Berndt et al., 2007; Tanedjeu et al., 2016). However, the results were different from another study which indicated that the *Salmonella* burden in cecal contents was not affected by probiotic treatments while *Salmonella* infections in liver and spleen were reduced (Yang et al., 2014). The differences may be associated with the types of probiotics used, breed and age of chickens, as well as rearing environments.

IFN-γ is a Th1 cytokine that stimulates macrophages to secret oxidants with antimicrobial activities and is produced by natural killer cells and T-lymphocytes (Alam et al., 2002). In this study, *C. butyricum* significantly decreased SE-induced IFN-γ expression level, which was similar to the report that pretreatment of 1-day-old chickens with probiotics could significantly reduce IFN-γ expression level in *Salmonella* infection period (Chen et al., 2012).

IL-1β is a major mediator of inflammation in birds and mammals, primarily produced by monocytes, tissue macrophages, and enterocytes (Bar-Shira and Friedman, 2006). In this study, *C. butyricum* significantly decreased SE-induced IL-1β expression level, which was consistent with a previous report which showed that treating *Salmonella*-infected chicks with *Lactobacillus* strains could significantly down-modulate the expression level of IL-1β (Chen et al., 2012).

IL-8, as an important member of the chemokines, has chemotactic activity and shares similar structure to cytokines (Baggiolini et al., 1997). The results in this study showed that *C. butyricum* could significantly reduce mRNA level of IL-8, which was also observed in a previous report (Yi et al., 2016).

LPS-induced TNF-α factor, as one kind of vital indicator for evaluating inflammatory response in chickens, can produce the inflammatory response in chickens when infected with pathogens (Feng et al., 2016). In the present study, *C. butyricum* significantly decreased SE-induced the mRNA level of TNF-α in the liver, which was consistent with the report that *Lactobacillus rhamnosus* may decrease *Escherichia coli*-induced TNF-α expression level (Liu et al., 2016).

TLR4 plays an essential role in the innate immune response and hence is likely to be involved in young chickens at risk of *Salmonella* infection (Li et al., 2010). In the study, *C. butyricum* suppressed inflammation by down-regulating TLR4, MyD88, and NF-κB-dependent pathways in chickens with SE infection on day 6 post-infection, which is consistent with the report that indicated that probiotics can decrease pro-inflammatory cytokine levels by inhibiting the expression of TLR4, MyD88, and NF-κB-dependent pathways in LPS-induced macrophages and in mice (Song et al., 2015; Yi et al., 2015).

Although there was a limitation in this study (the 2-week rearing period of SE infection experiment was relatively short), these findings indicated that *C. butyricum* can decrease SE infection by down-regulating cytokine gene expression, and can inhibit inflammation by down-regulating TLR4, MyD88, and NF-κB-dependent pathways. Collectively, *C. butyricum* could be a potential probiotics against SE infection in broiler chickens.

## Author Contributions

SS, HL, and XZ designed the work. XZ, JY, and LW raised animals. XZ and JY collected samples. XZ analyzed and interpreted data. XZ drafted the article. SS and HL critically reviewed the article.

## Conflict of Interest Statement

The authors declare that the research was conducted in the absence of any commercial or financial relationships that could be construed as a potential conflict of interest.
